# DNA Double-Strand Breaks: A Double-Edged Sword for Trypanosomatids

**DOI:** 10.3389/fcell.2021.669041

**Published:** 2021-04-15

**Authors:** Marcelo Santos da Silva

**Affiliations:** DNA Replication and Repair Laboratory (DRRL), Department of Chemical and Biological Sciences, Institute of Biosciences, São Paulo State University (UNESP), Botucatu, Brazil

**Keywords:** DNA double-strand breaks, homologous recombination, DNA repair, *Trypanosoma brucei*, *Trypanosoma cruzi*, *Leishmania* spp.

## Abstract

For nearly all eukaryotic cells, stochastic DNA double-strand breaks (DSBs) are one of the most deleterious types of DNA lesions. DSB processing and repair can cause sequence deletions, loss of heterozygosity, and chromosome rearrangements resulting in cell death or carcinogenesis. However, trypanosomatids (single-celled eukaryotes parasites) do not seem to follow this premise strictly. Several studies have shown that trypanosomatids depend on DSBs to perform several events of paramount importance during their life cycle. For *Trypanosoma brucei*, DSBs formation is associated with host immune evasion via antigenic variation. In *Trypanosoma cruzi*, DSBs play a crucial role in the genetic exchange, a mechanism that is still little explored but appear to be of fundamental importance for generating variability. In *Leishmania* spp., DSBs are necessary to generate genomic changes by gene copy number variation (CNVs), events that are essential for these organisms to overcome inhospitable conditions. As DSB repair in trypanosomatids is primarily conducted via homologous recombination (HR), most of the events associated with DSBs are HR-dependent. This review will discuss the latest findings on how trypanosomatids balance the benefits and inexorable challenges caused by DSBs.

## Introduction

DNA, the storage center of all genetic information of an organism, is continually assaulted by endogenous and exogenous sources of instability, resulting in a variety of possible injuries. Of these lesions, DNA double-strand breaks (DSBs) are the most threatening. If left unrepaired, DSBs drive genomic instability leading to cell death, and if repaired incorrectly, DSBs can drastically alter the genomic structure, for example, generating chromosomal translocations and rearrangements, both of which contribute to tumorigenesis in metazoans (Kaye et al., 2004; Cannan and Pederson, 2016; Zhao et al., 2020).

In general, endogenous DSBs can arise from metabolic reactions or DNA stressors. For instance, endogenous DSBs can arise during the attempted repair of oxidized DNA bases when they occur simultaneously on opposing strands (Yang et al., 2004; Cannan et al., 2014); or during DNA replication when the replication machinery encounters natural impediments that lead to pausing or blocking of the replication fork (Mirkin and Mirkin, 2007; García-Muse and Aguilera, 2016; da Silva et al., 2019); or during the processing of spontaneous single-stranded DNA breaks (SSBs) generated in the S-phase (Vilenchik and Knudson, 2003; Saleh-Gohari et al., 2005; Elango et al., 2017). Exogenous DSBs are generated predominantly by chemical mutagens or ionizing radiation (Cannan and Pederson, 2016; Carofiglio et al., 2018). Chemical mutagens usually include anticancer chemotherapeutic drugs, such as cross-linking agents (e.g., cisplatin), and radiomimetic compounds (e.g., phleomycin) (Chen and Stubbe, 2005; Wyrobek et al., 2005; Jekimovs et al., 2014). Ionizing radiation (IR) is a source of DSBs, but also SSBs following the production of radiolysis radicals that attack the sugar-phosphate backbone (Ward, 1994; Ma et al., 2012). In short, DSBs are often terminal lesions induced by a wide range of genotoxic conditions that, if unresolved, underpin genomic instability in eukaryotic cells.

DNA double-strand breaks have likely exerted pressure throughout eukaryotic evolution, selecting organisms that had developed a network of pathways and factors capable of efficiently dealing with this lesion (Xu and Price, 2011). The diversity of DNA repair pathways that exist and their conservation across the Eukarya domain support this hypothesis. Among conserved DNA repair pathways able to deal with DSBs are homologous recombination (HR), which requires the presence of a DNA template homologous to the damaged region, and the error-prone non-homologous end joining (NHEJ) pathway, which joins the DNA double-stranded ends in the absence of a homologous sequence (Farlow et al., 2011; Zhao et al., 2020).

Trypanosomatids (supergroup Excavata) have most of their DNA repair pathways conserved. However, notable divergencies exist suggesting a parasite-specific repurposing of the DSBs repair machinery (Glover et al., 2013; Ubeda et al., 2014; Alves et al., 2018; Mehnert et al., 2021). While the HR repair pathway is conserved and functional (McCulloch and Barry, 1999; Glover et al., 2008; Hartley and McCulloch, 2008; Genois et al., 2012; Alves et al., 2018; Marin et al., 2018), canonical NHEJ activities appear absent in trypanosomatids (Burton et al., 2007; Nenarokova et al., 2019). Instead, alternative NHEJ (Alt-NHEJ) pathways (e.g., microhomology-mediated end joining – MMEJ) and single-strand annealing (SSA) predominate to repair chromosomal DSBs in some trypanosomatid species (Glover et al., 2008; Peng et al., 2015; Rose et al., 2020). Several species of trypanosomatids are obligate parasites and can cause human diseases of great medical importance, including *Trypanosoma brucei* (*T. brucei*), *Trypanosoma cruzi* (*T. cruzi*), and *Leishmania* spp. These pathogens present a dixenous life cycle, i.e., perform stages of their life cycle in invertebrate and vertebrate hosts (Barratt et al., 2017). To survive and replicate inside their hosts, these organisms must overcome several barriers, including host defense mechanisms and unfavorable environmental conditions (Geiger et al., 2016). Intriguingly, some trypanosomatids can bypass these barriers using recombination events (Beverley et al., 1984; Myler et al., 1984b; Downing et al., 2011; Alves et al., 2018), which in many organisms, can be triggered following a DSB and its subsequent repair (Baudat and Nicolas, 1997; Pâques and Haber, 1999; Pfeiffer et al., 2000; Kuzminov, 2011). Trypanosomatids exploit their DSBs repair pathways and use them to their advantage to survive within a host. Thus, a fine-tuned balance must exist in these organisms to both facilitate the action of pathways DSBs-related and prevent repair machinery from being overwhelmed, which would compromise organism fitness.

In this review, I will discuss this paradoxical effect by which DSBs can act as opportunities for fundamental survival and adaptation mechanisms or as sources of genome instability in trypanosomatids.

## The Role of DSBs in the Evasion of *T. brucei* From the Host Immune Clearance

*Trypanosoma brucei* parasites cause debilitating and life-threatening conditions in mammals, including the African trypanosomiasis in humans and nagana in livestock. These infections persist due to the parasite’s ability to undergo antigenic variation. For *T. brucei*, this involves the stochastic switching of variant surface glycoproteins (VSGs), which hinders recognition and eradication mediated by the host immune system (Barry and McCulloch, 2001; Horn, 2014; Pinger et al., 2017; Ridewood et al., 2017).

Although the precise number of VSG genes that can encode a coat is unknown, around 2500 VSG-encoding genes have been cataloged in the nuclear genomes of *T. brucei* (Berriman et al., 2005; Cross et al., 2014; Müller et al., 2018). VSGs are located in the subtelomeric regions of the 11 diploid megabase chromosomes and also in the ∼ 100 mini and intermediate chromosomes (Wickstead et al., 2004; Marcello and Barry, 2007). Only one VSG is expressed at one time (i.e., expression is monoallelic) with the active VSG being transcribed from one of ∼15 dedicated telomere-proximal bloodstream expression sites (BESs) distributed among the 11 megabase chromosomes (Hertz-Fowler et al., 2008). Moreover, the activated BES is transcribed exclusively by RNA polymerase I (pol I) from an extranucleolar focus known as Expression Site Body (ESB) (Navarro and Gull, 2001). Recent studies have been demonstrating the strictness of the VSG expression control and how the ESB structure is important for active BES transcription (Kerry et al., 2017; Budzak et al., 2019; Faria et al., 2021).

In general, there are two main mechanisms by which a VSG gene can be switched (Myler et al.,1984a,b). The first one is called transcriptional switching and is characterized by alternating the subtelomeric region containing the ES that is being transcribed (Bernards et al., 1984; Myler et al.,1984a,b). The second involves different types of recombination events to perform VSG gene replacement, i.e., VSG genes can be shuttled from other locations in the chromosomes into an active ES (Myler et al., 1984b; Scholler et al., 1989; McCulloch et al., 1997; Conway et al., 2002; Hall et al., 2013). The VSG switching by recombination events predominates over transcriptional switching because it is possible to have access to the entire VSG repertoire through this mechanism (Robinson et al., 1999; Hovel-Miner et al., 2012). On the other hand, although frequently observed (Bernards et al., 1984; Myler et al., 1984a; Bitter et al., 1998; Rudenko et al., 1998; Barry and McCulloch, 2001), transcriptional switches allow access to only ∼15 VSGs housed in the ES. Recombination is also essential in the segmental gene conversion for generating antigenic diversity, producing a “VSGs mosaic” during chronic infections (Hall et al., 2013; Mugnier et al., 2015).

**FIGURE 1:**
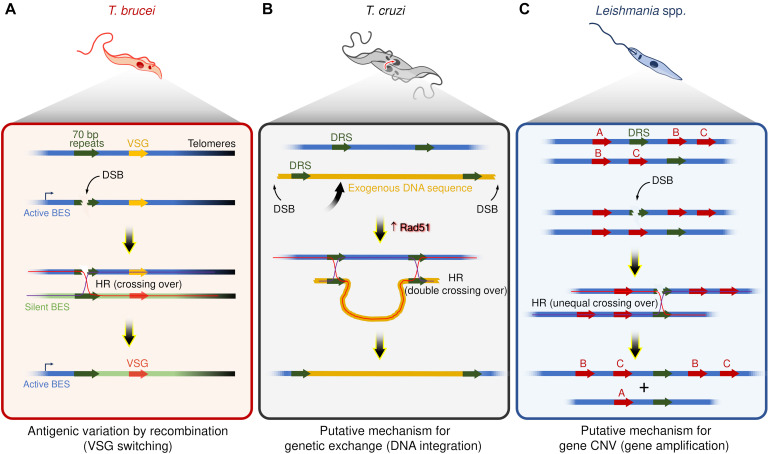
Examples of putative mechanisms dependent on recombination events and DSBs in trypanosomatids. **(A)** Antigenic variation by recombination events in *T. brucei* – DSBs within repeat elements (70 bp repeats) are catalysts for VSGs switching. Of note, DSBs occur naturally in active bloodstream expression sites (BES). **(B)** Genetic exchange in *T. cruzi* – Hybrid *T. cruzi* cells have slightly increased Rad51 expression (Alves et al., 2018), which may contribute to driving homologous recombination (HR) between direct repeated sequences (DRS) during the genetic exchange, resulting in the integration of an exogenous DNA. **(C)** Gene amplification in *Leishmania* spp. – DSBs nearby or within DRS may trigger HR and lead to gene copy number variation (gene CNV). In the scheme, the genes B and C were amplified.

At least two commonalities between recombination-based VSG switching and DSBs repair by HR strongly support that DSBs are catalysts for switch events (Figure 1A). First, the recombination-based VSG switching can be directly activated by the induction of a DSB in the active BES using the meganuclease I-*Sce*I (Boothroyd et al., 2009; Glover et al., 2013). Second, disruption of the HR pathway through the interruption of some components, such as ATR (Stortz et al., 2017; Black et al., 2020; Marin et al., 2020), Rad51 (McCulloch and Barry, 1999; Proudfoot and McCulloch, 2005), Rad50 (Mehnert et al., 2021), and BRCA2 (Hartley and McCulloch, 2008), impairs the VSG switching by recombination, suggesting that multiple components are shared between these two pathways. In general, these features mirror targeted gene rearrangements in other organisms, such as VAR genes diversity in *Plasmodium* (Kyes et al., 2007; Claessens et al., 2014), pilin antigenic variation in *Neisseria* (Cahoon and Seifert, 2011), and V(D)J recombination during the development of B lymphocytes of the vertebrate immune system (Tonegawa, 1983; Brecht et al., 2020).

Briefly, while DSBs may be potentially lethal according to the number, location, and DNA repair capacity of the cell (Marin et al., 2018), this DNA lesion is also a critical factor in the fundamental immune evasion mechanism carried out by *T. brucei*.

## DSBs Are Required During Genetic Exchange Performed by *T. cruzi*

*Trypanosoma cruzi* is the etiological agent of American trypanosomiasis (also known as Chagas disease), a potentially life-threatening illness afflicting ∼10 million people, predominantly across the Americas (Khare et al., 2016; Browne et al., 2017). Chagas disease encompasses a wide range of clinical manifestations during acute and chronic phases, such as viral−like symptoms (fever, malaise, and lymphadenopathy), arrhythmias, and transient electrocardiogram abnormalities (Morgan et al., 1996; Malik et al., 2015). Most of these symptoms are related to environmental factors and the broad genetic diversity presented by *T. cruzi* genetic groups (Andrade et al., 2002), of which six discrete typing units (DTUs), TcI to TcVI have been reported (Marcili et al., 2009; Zingales et al., 2012; Brenière et al., 2016).

A pervasive view is that *T. cruzi* proliferates by binary fission and subsequent clonal expansion (Tibayrenc et al., 1990; Ramírez and Llewellyn, 2014). However, in the last two decades, a growing number of studies support the existence of genetic exchange and possible cryptic sexual cycles among different populations of *T. cruzi* (Gaunt et al., 2003; Ramírez et al., 2012; Messenger and Miles, 2015; da Silva et al., 2018; Schwabl et al., 2019). For instance, although the evolutive relationships among the different DTUs are largely unclear, at least two DTUs (TcV and TcVI) are hybrids (Machado and Ayala, 2001; Pedroso et al., 2003; Sturm et al., 2003; Lewis et al., 2011; Messenger and Miles, 2015), evidencing that genetic exchange among distinct *T. cruzi* groups occurs naturally. Intriguingly, naturally occurring hybrid strains of *T. cruzi*, such as CL Brener (TcVI), show alterations in the expression of core HR factors, displaying high levels of BRCA2 and Rad51 transcripts, indicating that HR repair and DSBs could act as drivers of genetic exchange in these parasites (Alves et al., 2018; Figure 1B).

Unusually, *T. cruzi* displays remarkable resistance to ionizing radiation (IR), tolerating radiation exposure levels 50–100 times that of mammalian cells (Yonetani et al., 2005; Regis-da-Silva et al., 2006), an effect attributed to Rad51 directed activities acting to resolve IR-induced DSBs (Regis-da-Silva et al., 2006; Silva et al., 2018; Repolês et al., 2020). Indeed, *T. cruzi* appears to possess an extreme capacity to repair putative DSBs (Regis-da-Silva et al., 2006). Such capabilities could explain, in part, the ability of *T. cruzi* to produce hybrid strains. Perhaps unsurprisingly, *T. cruzi* populations overexpressing Rad51 also accumulate a high percentage of fused-cell hybrids (Alves et al., 2018), where Rad51 both acts to limit the formation/stabilization of fused-cell hybrids and drive HR events during the genetic exchange. *T. cruzi* hybrid strains appear better adapted to deal with DSBs relative to non-hybrid strains (Regis-da-Silva et al., 2006; Garcia et al., 2016; Cerqueira et al., 2017; Resende et al., 2020). This adaptation is probably related to an efficient HR pathway since Rad51 overexpression or ablation causes significant changes in how *T. cruzi* deals with DSBs (Regis-da-Silva et al., 2006; Silva et al., 2018).

Furthermore, some studies have been evidencing DSBs as a platform to facilitate other fundamental survival mechanisms, such as increased infectivity (Silva et al., 2018; Repolês et al., 2020), chromosome/gene copy number variation (Reis-Cunha et al., 2015), and variability in multigene families (Chiurillo et al., 2016). The latter is worth highlighting for lead to evasion of host immune response, a strategy like those used by *T. brucei* through antigenic variation (Myler et al., 1984b; Mugnier et al., 2015). Interestingly, the authors used the meganuclease I-*Sce*I to introduce programmed DSBs into a subtelomeric region of *T. cruzi* CL Brener (TcVI) and observed that the lesions were predominantly repaired by the Rad51-dependent mechanism: HR (Chiurillo et al., 2016). Whether other non-hybrid *T. cruzi* strains would repair programmed DSBs by HR is an issue that requires further investigation.

In conclusion, although multiple DSBs are harmful (Regis-da-Silva et al., 2006; Silva et al., 2018; Resende et al., 2020), *T. cruzi* likely utilizes these lesions to enable an increase in its genome diversity, a feature enhanced by Rad51. However, this raises an intriguing question: what did the naturally high levels of Rad51 expression lead to? The high tolerance to DSBs or the genetic exchange producing fused-cell hybrids? Considering that DSBs can trigger HR-dependent events (Pfeiffer et al., 2000; Li, 2015), and HR plays a crucial role in the genetic exchange (Alves et al., 2018), there will probably never be a satisfactory answer to this question.

## The Contribution of DSBs to Genomic Changes in *Leishmania* spp.

*Leishmania* spp. cause a spectrum of debilitating diseases collectively known as leishmaniasis, which have three main forms: visceral leishmaniasis (also known as kala-azar), which is characterized by the enlargement of the spleen and liver, concomitant with anemia and weight loss; cutaneous leishmaniasis, which causes skin lesions leaving serious disability or stigma; and mucocutaneous leishmaniasis, which destroy the mucous membranes of the nose, mouth, and throat (Nazzaro et al., 2014; Burza et al., 2018). To date, only a few human vaccines are in the clinical trial (Moafi et al., 2019), and parasite resistance to front-line drugs has been documented (Croft and Olliaro, 2011; Perez-Franco et al., 2016; Patino et al., 2019), making leishmaniasis a major global health problem.

*Leishmania* spp. have remarkably plastic genomes, with genomic alterations such as aneuploidy (Mannaert et al., 2012; Lachaud et al., 2014), and CNVs (Rogers et al., 2011; Bussotti et al., 2018), which seems to be widespread phenomena among the species (Rogers et al., 2011; Lachaud et al., 2014; Negreira et al., 2020). Interspersed throughout the genome of *Leishmania* spp. are repeated DNA sequence elements, which catalyze DNA rearrangements via the formation of circular and linear sequence amplicons (Beverley, 1991; Ubeda et al., 2014). These amplicons arise in several *Leishmania* spp. under stress conditions or when parasites are challenged with drugs (Beverley et al., 1984; Downing et al., 2011; Laffitte et al., 2014). As HR factors (e.g., Mre11, Rad50, BRCA2, and Rad51 paralogs) facilitate gene rearrangements (Grondin et al., 1993; Navarro et al., 1994; Genois et al., 2012, 2015; Laffitte et al., 2014; Ubeda et al., 2014), DSBs nearby or within repeat elements could act as initiators of amplicon formation (Figure 1C). Nevertheless, no studies have directly correlated DSBs with the emergence of extrachromosomal DNA elements or DNA rearrangement events in this organism to date. Despite this, DSBs are attractive substrates for this type of adaptive genome re-writing for at least three reasons: first, gene CNVs occur through rearrangements of repeated DNA sequences, a process that relies, at least partially, on HR (Grondin et al., 1993); second, increased expression of Rad51 is observed when DSBs are generated (McKean et al., 2001; Genois et al., 2012); and third, Rad51 inactivation prevents the formation of circular extrachromosomal elements even under drug pressure. However, linear amplicons can still form, suggesting that the production of circular extrachromosomal DNA elements is HR-dependent, whereas linear amplicon likely utilizes an alternative pathway (Ubeda et al., 2014; Genois et al., 2015).

Interestingly, more than half of the predicted extrachromosomal DNA elements in *Leishmania* spp. are present in wild-type populations in the absence of drug pressure indicating the *Leishmania* genome is, in fact, undergoing continuous rearrangement (Ubeda et al., 2014; Bussotti et al., 2018). Moreover, these stochastic rearrangements may reflect a strategy by which *Leishmania* can rapidly adapt to a changing environment (Ubeda et al., 2014). However, if DSBs are continually being generated to trigger these rearrangements is a matter of debate that remains open.

Succinctly, although multiple DSBs can be extremely hazardous for *Leishmania* spp. (Manna et al., 2010; da Silveira et al., 2013), further studies are necessary to finish the puzzle promoted by these lesions and find out when they can be a benefit or a detriment for this parasite.

## Discussion

Antigenic variation in *T. brucei*, genetic exchange in *T. cruzi*, and genomic alterations in *Leishmania* are examples of some vital processes triggered by DSBs and evidence how fundamental is this type of DNA damage for these organisms. However, some studies have shown that the response to DSBs can be slightly different in each trypanosomatid. For instance, *T. brucei* apparently fails to trigger a stringent cell cycle checkpoint in response to DSBs and, due to that, DNA breaks may persist during cell division until a template (e.g., sister chromatid) is available (Glover et al., 2019). This finding suggests that MMEJ does not play a major role in *T. brucei*. In contrast, DSBs generated by CRISPR/Cas9 without a template do not persist in *T. cruzi* and are repaired by MMEJ (Peng et al., 2015). Curiously, *Leishmania donovani* predominantly uses SSA instead of MMEJ to repair DSBs introduced by CRISPR/Cas9 (Zhang et al., 2020). These different behaviors in response to DSBs suggest that the cell cycle plays a fundamental role in the trypanosomatids DNA damage response.

In population terms, the cell cycle phase where DSBs are generated is trivial since the predominant phenotype is evidenced by those trypanosomatids that managed to overcome the DNA damage. However, for a single cell, the cell cycle phase in which DSBs are introduced is essential to decide its fate. For instance, DSBs generated outside the S/G2 phases are unlikely to trigger recombination events, mainly due to the absence of a sister chromatid (homologous sequence). This behavior may explain the different and peculiar responses to the DSBs previously mentioned. In this scenario, single-cell analyses (e.g., single-cell transcriptomics) can be a valuable tool to reveal possible cryptic populations capable of dealing with DSBs differently (Briggs et al., 2021). Profiling gene expression of individual cells with single-cell RNA sequencing may detect rare cell types in heterogeneous populations previously challenged with DSBs source agents, such as IR. This approach may contribute to evidence, even more, how relevant are the roles of DSBs in the life cycle of these peculiar organisms.

## Concluding Remarks

**FIGURE 2:**
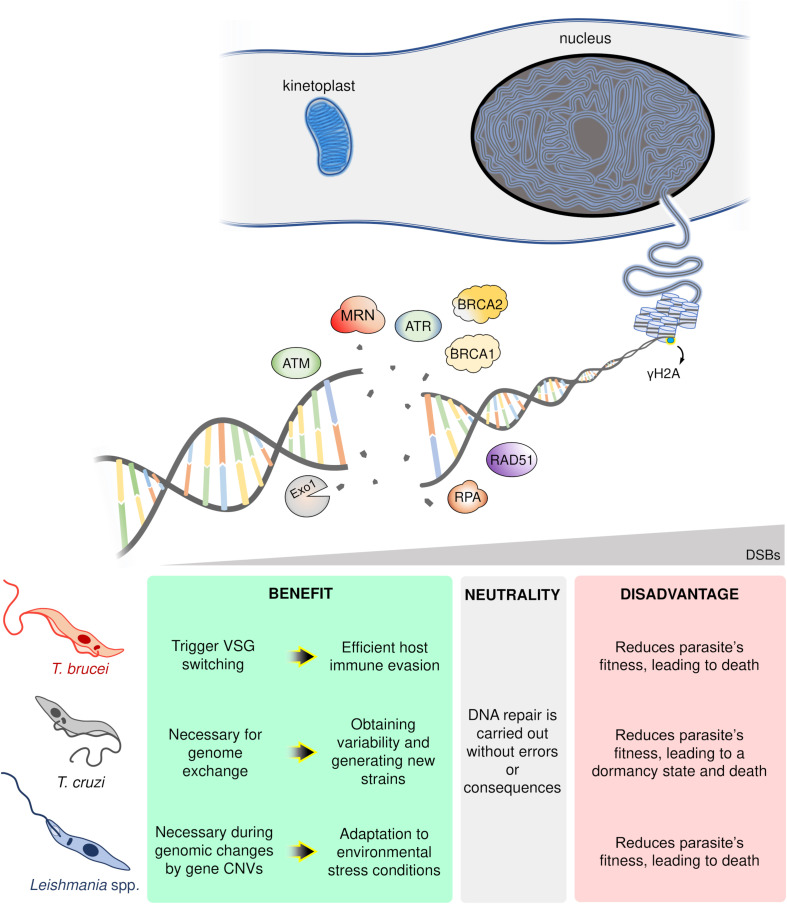
Schematic overview of the possible trypanosomatid cell’s fate in response to DNA double-strand breaks (DSBs). In a hypothetical trypanosomatid, several players act in an orchestrated way in response to DSBs. However, according to the number, location, cell cycle phase, and DNA repair capacity of the cell, these lesions can trigger different consequences: advantages (green box), neutrality (gray box), or disadvantages (red box). ATM, ataxia telangiectasia mutated; ATR, ataxia telangiectasia and Rad3-related; MRN, MRE11-RAD50-NBS1 complex; Exo1, Exonuclease 1; RPA, Replication protein A; BRCA1-2, Breast cancer 1–2; Rad51, Recombinase involved in homologous recombination; γH2A, phosphorylated histone H2A.

In conclusion, DSB formation poses a conundrum for single-celled organisms like trypanosomatids. On the one hand, DSBs undermine genomic stability compromising parasites fitness and potentially inducing death (Regis-da-Silva et al., 2006; Manna et al., 2010; Marin et al., 2018). On the other, DSBs provide an essential substrate for genome variability and subsequent adaptation to rapidly changing environments, with examples from each parasite harnessing DSBs and its repair to this effect: in *T. brucei*, DSBs can trigger VSG switching enabling host immune evasion (Boothroyd et al., 2009; Glover et al., 2013); for *T. cruzi*, DSBs are necessary for HR-dependent events essential for genetic exchange (Gaunt et al., 2003; Alves et al., 2018) and variability in multigene families (Chiurillo et al., 2016); and in the case of *Leishmania* spp., DSBs can be catalysts for recombination events leading to genomic changes and CNVs, a crucial strategy to overcome hostile environments (Grondin et al., 1993; McKean et al., 2001; Laffitte et al., 2016). Thereby, DSBs represent a “double-edged sword” for trypanosomatids (Figure 2). Now, further studies are required to establish which players (or pathways) wield this heavy blade.

## Author Contributions

MSdS wrote, revised, and approved the submitted version of the manuscript.

## Conflict of Interest

The author declares that the research was conducted in the absence of any commercial or financial relationships that could be construed as a potential conflict of interest.
